# Pediatric Patients in a Local Nepali Emergency Department: Presenting Complaints, Triage and Post-Discharge Mortality

**DOI:** 10.1177/2333794X20947926

**Published:** 2020-09-18

**Authors:** Samita Giri, Tine Halvas-Svendsen, Tormod Rogne, Sanu Krishna Shrestha, Henrik Døllner, Erik Solligård, Kari Risnes

**Affiliations:** 1 Norwegian University of Science and Technology, Trondheim, Norway; 2 Dhulikhel Hospital, Kathmandu University Hospital, Dhulikhel, Nepal; 3 St. Olav’s Hospital, Trondheim University Hospital, Trondheim, Norway

**Keywords:** pediatric, emergency, presenting complaints, mortality, Nepal

## Abstract

*Background.* In low-income countries, pediatric emergency care is largely underdeveloped although child mortality in emergency care is more than twice that of adults, and mortality after discharge is high. *Aim.* We aimed at describing characteristics, triage categories, and post-discharge mortality in a pediatric emergency population in Nepal. *Methods.* We prospectively assessed characteristics and triage categories of pediatric patients who entered the emergency department (ED) in a local hospital. Patient households were followed-up by telephone interviews at 90 days. *Results.* The majority of pediatric emergency patients presented with injuries and infections (~40% each). Girls attended ED less frequent than boys. High triage priority categories (orange and red) were strong indicators for intensive care need and for mortality after discharge. *Conclusion.* The study supports the use and development of a pediatric triage systems in a low-resource general ED setting. We identify a need for interventions that can reduce mortality after pediatric emergency care. Interventions to reduce pediatric emergency disease burden in this setting should emphasize prevention and effective treatment of infections and injuries.

## What do You Already Know About This Topic?

Emergency diseases or conditions contributes to 45% of all deaths and 36% of disease burden including disabilities in low resource countries, and there is a huge knowledge gap in pediatric emergency medicine in low-income countries.

## What Does Your Research Contribute to the Field?

Infections and injuries complaints dominates the pediatric emergency population, and high triage priority categories were strong indicators for intensive care need and for mortality after discharge.

## What Does Your Research’s Implications Toward Theory, Practice and Policy?

The present study indicates that the pediatric triage system performed well in predicting intensive care need and mortality when applied in a hospital in Nepal that was not a specialized pediatric emergency department. This support that pediatric triage systems can be effectively implemented in similar low resource settings, that are not pediatric specialized centers. The study also indicates a high post-discharge and that there is need to develop interventions that can reduce mortality after pediatric emergency care.

## Introduction

More than 6 million children under 15 years died worldwide in 2017, 5.4 million of them were under the age of 5 years.^1^ UNICEF reported that 80% of under-5 mortality were in South Asia and Sub-Sahara Africa.^2^ More than half of these deaths could be prevented and treated with low-cost interventions.^1-3^

It is warranted that pediatric emergency health care services are addressed to effectively reduce child mortality.^4,5^ The Disease Control Priorities project has estimated that almost 45% of deaths and 36% of disability-adjusted life years (DALYs) in low and middle income countries (LMICs) are compounded with diseases and injuries that need to be addressed by emergency health care services.^6^ The situation for pediatric emergency care seems to be a particular target for improvements^4^: A systematic review by Obermeyer et al. from emergency departments (EDs) in 59 LMICs reported an overall median mortality of 1.8%, and mortality was much higher in pediatric facilities (4.8%) compared to adult or general facilities (0.7%).^7^ The most recent “Global Burden of Disease Study” reported an alarming sepsis incidence among children <5 years (41.5%) with 26.3% sepsis deaths in these age group.^8^ In line with this, the World Health Organization (WHO) has concluded that strengthening pediatric capacity and competence in health systems would effectively reduce many unwanted child deaths.^1^ However, pediatric emergency care is typically underdeveloped in LMICs. Nepal is one such example: Although under-5 child mortality is unacceptably high (39 deaths per 1000 live births),^9^ there is no system for pediatric emergency care.^10^ A remarkable knowledge gap in pediatric emergency epidemiology in LMICs makes appropriate planning of emergency services challenging.^7,11^ Pediatric post-discharge mortality (PPDM) in developing countries is high, and a systematic review by Nemetchek et al. concluded recently that PPDM occurs in similar numbers or exceeds the in-hospital mortality.^12^ To reduce child mortality, one must therefore emphasize interventions that can reduce both in-hospital mortality and PPDM. We have previously described characteristics and post-discharge mortality among adults in a Nepalese hospital.^13^ The post-discharge mortality in adult population was more than 20-fold the ED mortality, and was particularly high among patients with respiratory and cardiovascular complaints. Similar studies relevant to pediatric emergency care are scarce.

The present study aims to describe characteristics, presenting complaints (PCs) and triage categories in a pediatric emergency population and to explore associations with post-discharge mortality. Thus, in a subset of the population, follow-up information was assessed by telephone interviews with family members 90 days after discharge from emergency care.

## Materials and Methods

### Study Design and Setting

A prospective observational study was conducted in the ED of Dhulikhel Hospital (DH), a 375 bedded non-government university hospital in Nepal. The hospital has a neonatal and a pediatric intensive care unit, and a pediatric ward with 45 beds in total. This hospital is located in semi-urban region in Dhulikhel, in Kavrepalanchok district 30 km northeast of Kathmandu.

Kavrepalanchok district has a total population of nearly 400 000, of them 51% are female.^14^ The median age in this region is 23 years, and 20% are 0 to 15 years old.^14^ The three main ethnic groups in the district are Brahmin or Chhetri (36%) followed by Janajati (51%) and Dalit (7%).^14^ The living conditions in Kavrepalanchok district are generally quite basic; one example is that 78% of the population use wood as a main type of cooking fuel.^14^ Based on 2017 death registry of the district, the total mortality of the region is 1.2%.

### Data Collection and Participants

Demographic and clinical information was prospectively registered on systematic emergency forms by ED staffs for all patients who sought care at the ED from September 2013 to December 2016 (Supplementary Material). Patients 16 years and under were included in the current study (Figure 1A). Data collection was interrupted by infrastructure challenges (Sept 2014-Feb 2015) and (Sept and Nov 2016), and earthquakes (April 25-May 16, 2015). Data from the earthquake period has been described previously.^15^

**Figure 1A.**
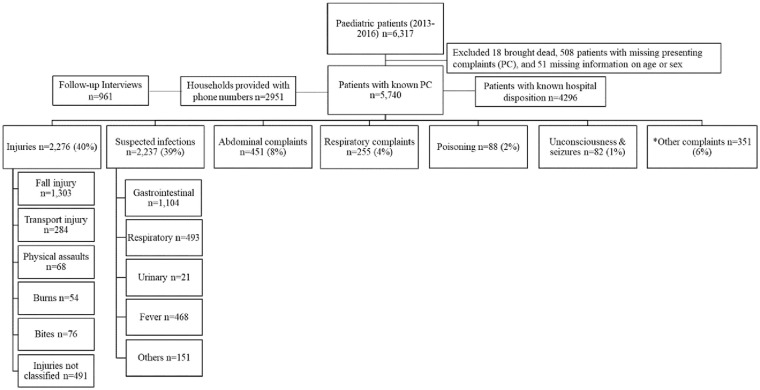
Flow diagram of cohort and distribution of presenting complaints. Abbreviation: PC, presenting complaint. *Other complaints included musculoskeletal, neurology, urinary, cardiovascular diseases or complaints, psychology, and other general complaints.

The pediatric version of Rapid Emergency Triage and Treatment System (RETTS-P)^16^ with 4 color codes: red, orange, yellow, and green for very high, high, medium, and low risk, respectively was used to assess all pediatric patients at presentation in the ED from March 2015 to April 2016. We used the English version of RETTS-P. RETTS was developed in Sweden and has been increasingly used in the Scandinavian countries. RETTS-P has been described in previous studies and reliability has been reported as good.^17-19^ RETTS-P has not been reported used in low-resource settings. Patients that were triaged and were eligible for the telephone interviews (Figure 1B) were further assessed for mortality at 90 days.

**Figure 1B.**
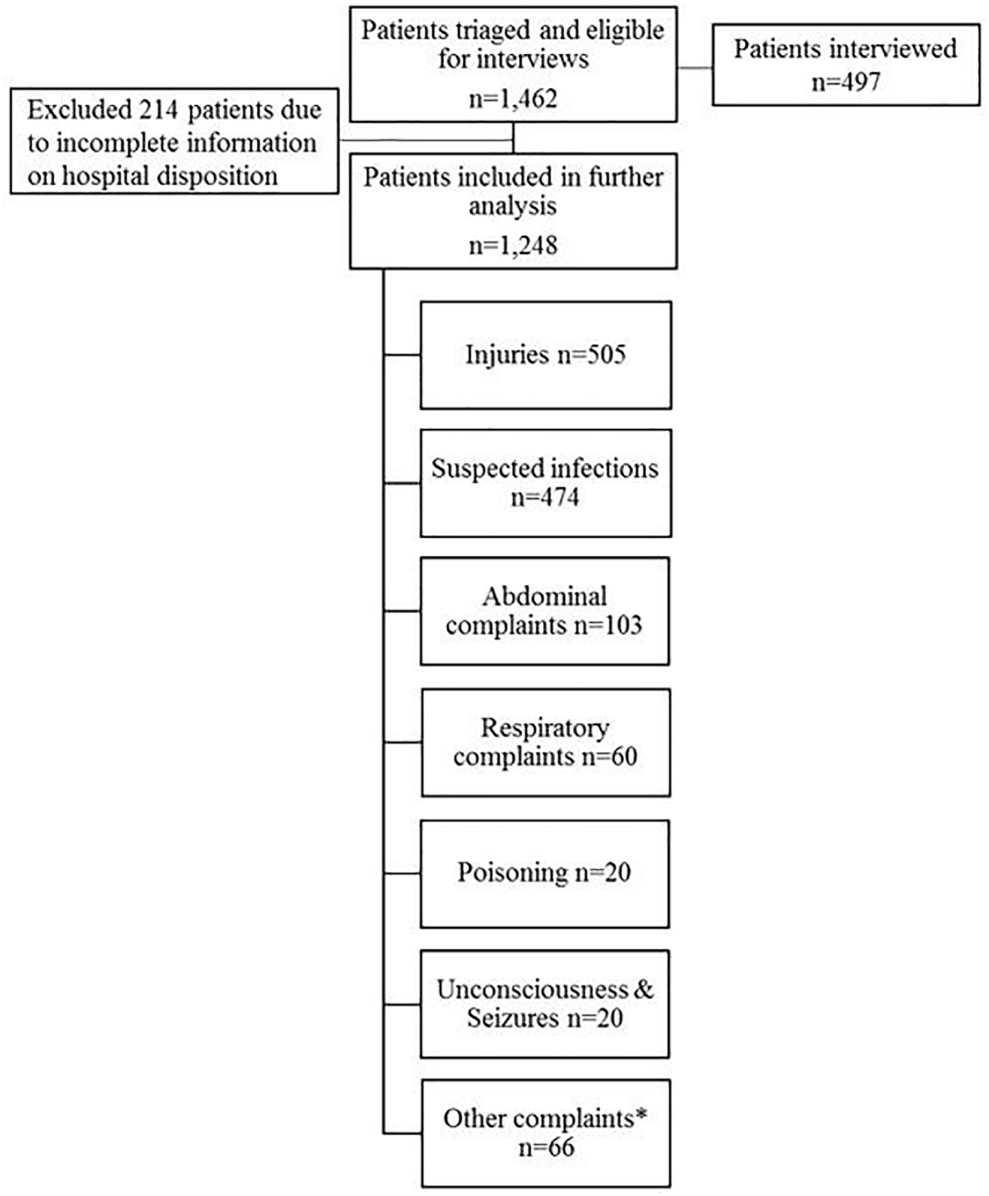
Flow diagram of triaged patients and interviewed. *Other complaints included musculoskeletal, neurology, urinary, cardiovascular diseases or complaints, psychology, and other general complaints.

Figures 1A and 1B shows the population and subsets of the population that were included in the analyses. We analyzed two sets of the population: First, we assessed all pediatric patients that were registered in the ED during the whole study period (Figure 1A). Next, we restricted our population to those who had arrived in ED after triage system for children had been introduced (Figure 1B).

### Follow-Up Interviews

At ED disposition, a family member was asked for consent for a telephone-interview at 90 days after initial presentation to the ED. Trained research nurses called those who consented and performed the structured telephone interview with the family members of pediatric patients. The structured questionnaire (Supplementary Material) included information on death during last 90 days and living conditions (use of traditional stove for cooking).

### Variables

Research nurses with detailed knowledge of the area used the patient’s home addresses to categorize their residence into rural (living outside a municipality) or urban (living inside a municipality). Ethnicity is closely related to socio-economic status in Nepal and was categorized into four groups recognized by Nepali authorities; Brahmin and Chhetri, Janajati, Dalit, and others. Brahmin and Chhetri are generally considered as a group having a more privileged socioeconomic status and Dalit typically have a less privileged socioeconomic status.^20^

Time of presentation at ED was categorized: daytime (08-16 weekdays) or after working hours (16-08, and holidays). ED disposition was categorized as follows: admission to pediatric or general wards; admission to intensive care unit (ICU), operating theater (OT), or referral to other hospitals; discharge from ED; death in ED. Admission to ICU or OT, and referral to other hospital were grouped together because these were considered in need of advanced care directly from the ED. Referrals from ED typically constitute those in need of intensive care or surgery when there was not enough capacity or when specific care was not available at study hospital (typically neurosurgery).

### Presenting Complaints Classification

The PCs from the emergency forms were translated into “International Classification of Primary Care-2 (ICPC-2)” codes,^21^ and classified into seven main categories; injuries, suspected infections, abdominal complaints, respiratory complaints, poisoning, unconsciousness and seizures, and other complaints (Figure 1A). The strategy for presenting complaint categorization and use of ICPC-2 codes is presented in the Supplemental Material Table S1.

### Data Analysis

Data analyses were performed using STATA 15 (StataCorp LP, College Station Texas, USA). Descriptive data is presented by numbers and percentages. Associations between patient characteristics and outcome variables (mortality at 90 days and ED disposition) were assessed by logistic regression. Unadjusted odds ratios (ORs) and ORs adjusted for age in years and sex (aOR) are presented with 95% confidence intervals (CIs).

Sensitivity analyses were performed to assess possible selection bias related to those that could be followed for interviews after discharge (Tables S2-S6 in Supplementary Material). Thus, we assessed whether characteristics in the subpopulations who could be followed by interview and those that were assessed by triage were different from those who were not included in these analyses by comparing distributions of characteristics in these groups. We also assessed possible differences in population characteristics before and after the earthquake period (Table S7 in Supplementary Material).

### Ethical Approval and Informed Consent

The study was approved by the institutional ethical review committee of Kathmandu University School of Medical Sciences in Nepal (approval number 58/13) and the Regional Committee for Medical and Health Research Ethics in South East Norway (approval number 2014/1246). As this study is based on routinely collected pseudo anonymized patient information in the hospital, informed consent from the patients was not obtained in individuals, as approved by the local ethical committee. Verbal consent was taken for information on telephone numbers, and at the beginning of the telephone interviews.

## Results

A total of 6317 patients presented to ED, of which 2591 consented to call, and 961 were eventually followed up (Figure 1A). The triaged population numbered 1462 children: 1248 (85%) of these had complete information and 497 (34%) were telephone interviewed 90 days after discharge (Figure 1B).

Analyses comparing characteristics of those included and not included in the sub populations are presented in Supplemental Tables S2 to S6. Overall, these analyses show no meaningful differences between those included in the follow-up analyses and those who could not be followed. Likewise, the analysis of characteristics comparing those before and after the earthquakes in Table S7 show no significant differences in patient characteristics and hospital mortality after emergency care (mortality data not reported in publication).

The distribution of presenting complaints and demographic variables according to the main categories of presenting complaints are presented in Table 1. The median age of the population was 7 years, and 61% were 5 years or older. Boys were overrepresented in the total ED population; 63% of the children were male, and this proportion was similar in most presenting complaints groups. The children typically lived in rural areas (61%), and with similar distributions for rural living within the PC groups. The distribution of ethnicity was similar for the different PC groups, and typically 40%-50% of the children were from the more privileged groups Brahmin and Chhetri. The PCs were dominated by injuries and infections, each accounting for ~40%. Children with infection were typically younger (median 4 years) than injured children (median 9 years).

**Table 1.** Baseline Characteristics and Emergency Department Disposition by Categories of Presenting Complaints in 5740 Children Presenting to Emergency Department in Dhulikhel Hospital from Sept 2013 to Dec 2016.

Characteristics	Total	Injuries	Suspected infections^Ϯ^	Abdominal complaints	Respiratory complaints	Poisoning	Unconsciousness & seizures	Other complaints^ǂǂ^
**Total Patients, n (%)**	**5740**	**2276 (40)**	**2237 (39)**	**451 (8)**	**255 (4)**	**88 (2)**	**82 (1)**	**351 (6)**
**Age, (years) median (IQR)**	7 (2-12)	9 (5-13)	4 (1-10)	11 (5-15)	2 (1-9)	5 (2-14)	13 (6-15)	7 (1-14)
**Age (years), n (%)**								
<1	521 (9)	26 (1)	319 (14)	36 (8)	68 (27)	4 (5)	1 (1)	67 (19)
1-<5	1740 (30)	506 (22)	936 (42)	68 (15)	95 (37)	39 (44)	18 (22)	78 (22)
5-16	3479 (61)	1744 (77)	982 (44)	347 (77)	92 (36)	45 (51)	63 (77)	206 (59)
**Sex, n (%)**								
Female	2110 (37)	718 (32)	870 (39)	183 (41)	101 (40)	40 (45)	54 (66)	144 (41)
Male	3630 (63)	1558 (68)	1367 (61)	268 (59)	154 (60)	48 (55)	28 (34)	207 (59)
**Patient location, n (%)**								
Rural	3522 (61)	1499 (66)	1304 (58)	256 (57)	144 (56)	54 (61)	53 (65)	212 (60)
Urban	1709 (30)	566 (25)	757 (34)	165 (37)	81 (32)	27 (31)	16 (20)	97 (28)
Information NA	509 (9)	211 (9)	176 (8)	30 (7)	30 (12)	7 (8)	13 (16)	42 (12)
**Ethnicity, n (%)**								
Brahmin and Chhetri	2573 (45)	944 (41)	1072 (48)	222 (49)	113 (44)	33 (38)	40 (49)	149 (42)
Janajati	2546 (44)	1068 (47)	932 (42)	193 (43)	117 (46)	44 (50)	31 (38)	161 (46)
Dalit	507 (9)	219 (10)	193 (9)	30 (7)	19 (7)	8 (9)	9 (11)	29 (8)
Other	114 (2)	45 (2)	40 (2)	6 (1)	6 (2)	3 (3)	2 (2)	12 (3)
**Presentation to ED, n (%)** *								
08:00-16:00 weekdays	1037 (24)	481 (28)	322 (19)	96 (28)	37 (20)	23 (38)	24 (40)	54 (22)
16:00-08:00 or holidays	3259 (76)	1258 (72)	1337 (81)	247 (72)	151 (80)	37 (62)	36 (60)	193 (78)
**ED disposition, n (%)** *								
Pediatric/other ward	1267 (29)	465 (27)	567 (34)	76 (22)	56 (30)	19 (32)	17 (28)	67 (27)
ICU or OT or Referred	341 (8)	126 (7)	117 (7)	26 (8)	26 (14)	18 (30)	7 (12)	21 (9)
Discharged	2682 (62)	1145 (66)	975 (59)	241 (70)	105 (56)	23 (38)	35 (58)	158 (64)
Died in ED	6 (0.1)	3 (0.2)	0	0	1 (0.5)	0	1 (2)	1 (0.4)

Abbreviations: IQR, inter quartile range; NA, not available; ED, emergency department; ICU, intensive care unit; OT, operation theater.

Ϯ Suspected infections and fever.

ǂǂ Other complaints included musculoskeletal, neurology, urinary, cardiovascular diseases or complaints, psychology, and other general complaints.

* n = 4296 (75%) has complete information and included in the analysis.

Roughly 80% of children presented in the ED after office hours and during holidays (Table 1). Over 60% were discharged home without hospitalization, and 8% were admitted directly from ED to ICU or OT or were referred to other health facility. Less than 1% died in the ED. The patterns of demographic characteristics and disposition were similar for most PC groups. However, among unconscious and poisoned children some different patterns were displayed. There was a larger proportion of girls than boys who were brought unconscious, and 30% of poisoned children were admitted directly to higher levels of care.

Results for the population that was interviewed 90 days after discharge from ED is shown in Table 2. Post-discharge mortality was low; only 12 (1.3%) of the 961 interviewed patients had died. Risk of PPDM was higher in children under 1 year compared to children 5-16 years, (sex-adjusted OR 4.3, 95% CI = 1.2-15.5). Also, there was a tendency toward higher risk of PPDM in girls (2.1%) than boys (0.8%), (age-adjusted OR 2.6, 95% CI = 0.8-8.4)). Mortality tended to be higher in rural populations compared with urban populations (age and sex adjusted (aOR) 5.9, 95% CI = 0.8-46.5). Children from homes with a traditional stove had an increased mortality risk (aOR 13.8, 95% CI = 1.8-108.3). When assessing mortality in different PC categories, 2 out of 3 PPDM deaths had presented as suspected infections (aOR for mortality after infections compared to other complaints was 2.7, 95% CI = 0.8-9.4).

**Table 2.** Association Between Patient’s Characteristics and Presenting Complaint Categories, and 90 Days Mortality Among 961 Children Interviewed by Telephone 90 Days After Emergency Department Visit from September 2013 to December 2016.

Characteristics	Total interviewed	90-days mortality	Unadjusted	Adjusted^¶^
			OR 95% CI	OR 95% CI
**Total, n (%)**	**961**	**12 (1.3)**		
**Sex, n (%)**				
Female	340 (35)	7 (2.1)	2.6 (0.8-8.2)	2.6 (0.8-8.4)
Male	621 (65)	5 (0.8)	1	1
**Age, (years), n (%)**				
<1	96 (10)	4 (4.2)	4.2 (1.2-15.3)	4.3 (1.2-15.5)
1-<5	275 (29)	2 (0.7)	0.7 (0.1-3.6)	0.7 (0.1-3.5)
5-16	590 (61)	6 (1.0)	1	1
**Patient location, n (%)**				
Urban	313 (33)	1 (0.3)	1	1
Rural	648 (67)	11 (1.7)	5.4 (0.7-41.9)	5.9 (0.8-46.5)
**Exposure to traditional stove**				
No	505 (53)	1 (0.2)	1	1
Yes	456 (47)	11 (2.4)	12.5 (1.6-96.9)	13.8 (1.8-108.3)
**Presenting Complaints, n (%)**				
Suspected infections	380 (40)	8 (2.1)	3.1 (0.9-10.4)	2.7 (0.8-9.4)
Other complaints	581 (60)	4 (0.7)	1	1
**ED disposition, n (%)** *				
General wards	298 (36)	2 (0.7)	1	1
ICU or OT or Referred	61 (7)	2 (3.3)	5.0 (0.7-36.3)	4.7 (0.6-34.5)
Discharged	466 (57)	6 (1.3)	1.9 (0.4-9.6)	1.9 (0.4-9.6)

Abbreviation: CI, confidence interval.

* Analysis done with smaller denominators.

¶ Adjusted for sex and age in continuous.

Results for the triaged population is shown in the Tables 3A to 3C. Distribution of triage categories by presenting complaint categories is shown in Table 3A. Overall, 4% of children had a red triage category, 15% orange, 49% yellow, and 32% green. Considering red and orange cases as high severity with need of urgent care, these 2 categories added up to 60% of the poisoned patients, 23% of the infections, 13% of the injured, and 14% of abdominal complaints. Within infections, suspected respiratory tract infections had the highest severity (triage categories red or orange) indicated by 32% (Table 3B).

**Table 3A.** Distribution of Triage Categories by Categories of Presenting Complaints Among Children Presenting to Emergency Department in Dhulikhel Hospital from March 2015 to April 2016.

Characteristics	Total	Injuries	Suspected infectionsϮ	Abdominal complaints	Respiratory complaints	Poisoning	Unconsciousness & seizures	Other complaints^ǂǂ^
**Total Patients, n (%)**	**1248**	**505 (40)**	**474 (38)**	**103 (8)**	**60 (5)**	**20 (2)**	**20 (2)**	**66 (5)**
**Triage (Yes), n (%)**								
Red	50 (4)	12 (2)	20 (4)	3 (3)	5 (8)	6 (30)	2 (10)	2 (3)
Orange	183 (15)	56 (11)	88 (19)	11 (11)	10 (17)	6 (30)	4 (20)	8 (12)
Yellow	617 (49)	218 (43)	266 (56)	57 (55)	30 (50)	8 (40)	11 (55)	27 (41)
Green	398 (32)	219 (43)	100 (21)	32 (31)	15 (25)	0	3 (15)	29 (44)

Ϯ Suspected infections and fever.

ǂǂ Other complaints included musculoskeletal, neurology, urinary, cardiovascular diseases or complaints, psychology, and other general complaints.

**Table 3B.** Distribution of RETTS Triage Categories by Categories of Infections Among Children Presenting to Emergency Department in Dhulikhel Hospital from March 2015 to April 2016.

Characteristics	Total	Gastrointestinal infections	Urinary infections	Respiratory infections	Fever	Other infections*
**Total Patients, n (%)**	**474**	**233 (49)**	**2 (0.4)**	**119 (25)**	**89 (19)**	**31 (7)**
**Triage, n (%)**						
Red	20 (4)	7 (3)	0	9 (8)	2 (2)	2 (6)
Orange	88 (19)	37 (16)	0	28 (24)	15 (17)	8 (26)
Yellow	266 (56)	134 (58)	0	63 (53)	52 (58)	17 (55)
Green	100 (21)	55 (23)	2 (100)	19 (16)	20 (22)	4 (13)

* Other infections included otitis media, skin infection, discharge from wound, and other infections.

**Table 3C.** Triage Priority Levels and Disposition (admission to ward, and admission to ICU or OT or referred to other hospitals) in the Emergency Department Among 1248 Children Presenting to Dhulikhel Hospital from March 2015 to April 2016.

Characteristics	Triage	Total	Ward vs **discharged**	Unadjusted	Adjusted^¶^	ICU/OT/Referred vs **Discharged**	Unadjusted	Adjusted^¶^
				OR 95% CI	OR 95% CI		OR 95% CI	OR 95% CI
**Total Patients, n (%)**		1248	294 (24)			83 (7)		
**Triage code n (%)** *	Red	50 (4)	14 (28)	4.1 (1.9-8.9)	4.1 (1.9-8.9)	21 (42)	33.8 (14.3-80.2)	32.1 (13.5-76.6)
	Orange	183 (15)	57 (31)	2.5 (1.7-3.8)	2.5 (1.6-3.7)	27 (15)	6.6 (3.3-13.3)	6.4 (3.2-12.8)
	Yellow	617 (49)	152 (25)	1.5 (1.1-2.1)	1.5 (1.1-2.0)	22 (4)	1.2 (0.6-2.4)	1.1 (0.6-2.3)
	Green	398 (32)	71 (18)	1	1	13 (3)	1	1

¶ Adjusted for sex and age in continuous.

* Percentages for total is column percentage and others are row percentage.

Associations between triage category and hospital disposition is shown in Table 3C. For red category, 28% were admitted to the general wards (aOR was 4.1, 95% CI = 1.9-8.9 compared to discharge from ED), and 42% were admitted to ICU or OT or were referred to other hospitals (aOR for the latter 42% was 32.1, 95% CI = 13.5-76.6 compared to discharge from ED).

Associations for PPDM in the interviewed population that had received a triage code is presented in table 4. Nearly 16% of those triaged red had died at time of interview and aOR for death in red category was 56.5 (95% CI = 4.6-687.3). Mortality in orange category (2.6%) was also high compared to green category (0.7%), but with low numbers and low precision.

**Table 4.** Triage Priority Levels and 90 Days Mortality Among 497 Children Presenting to the Emergency Department at Dhulikhel Hospital from March 2015 to April 2016.

Characteristics	Triage	Total	90-days mortality	Unadjusted	Adjusted^¶^
				OR 95% CI	OR 95% CI
**Total Patients, n (%)**		497	9 (1.8)		
**Triage code (n = 497), n (%)**	Red	19 (3.8)	3 (15.8)	28.5 (3.0-290.3)	56.5 (4.6-687.3)
	Orange	78 (15.7)	2 (2.6)	4.0 (0.4-44.8)	3.7 (0.3-43.2)
	Yellow	247 (49.7)	3 (1.2)	1.9 (0.2-18.1)	1.6 (0.2-16.1)
	Green	153 (30.8)	1 (0.7)	1	1

¶ Adjusted for sex and age in continuous.

## Discussion

Infections and injuries were dominating patient complaints in this pediatric emergency population. Urgency, identified by orange or red triage code at presentation, was particularly high in children with infections. Also, red triage code was strongly associated with more advanced level of care and increased post-discharge mortality risk.

### Strengths and Limitations

This is a single-center study; thus generalizations should be done with caution. However, the cohort comprised a large population from both rural and urban regions and the distribution of patient characteristics show that the patient population is highly representative for the region in respect to age, gender, geography, and ethnicity. Also, the long data collection period is a strength; the study includes data from a 3-year period, covering possible seasonal variations. However, it is a limitation that the study had low follow-up rates for the 90 days telephone interviews. Therefore, the results related to interview information should be interpreted with caution. However, again, we show that those lost to follow-up have similar characteristics and PCs to those who could be followed. We cannot rule out the possibility of selection bias in the interviewed patients, possibly healthier, and more resourceful families could be followed by telephone interview, which may lead to possible underestimation of mortality. Patients who did not provide their telephone number were not interviewed and these patients might not have had a phone due to economic conditions and could also be frailer than the ones available for interviews. Also, it is possible that families who were called and did not answer were more likely to have lost a child. It is a limitation that of the triage system RETTS-P was in use for only a part of the period. However, triage data was collected for almost a year and all data from this period was reported in the study. Importantly, RETTS triage system has never been reported from low income settings and the present study is the first scientific report to demonstrate usefulness of this system in a low-resource pediatric setting. Moreover, the current study does not include information related to specific diagnostic tools and treatments that will affect mortality.

### Characteristics of Patients

In our study injuries and suspected infections dominated the presenting complaints in children <16 year of age. We have identified 6 studies^22-27^ that have described the disease spectrum of pediatric emergency patients in Asian LMICs, and some patterns are remarkably similar across studies. Broadly, infections and injuries are dominating in unselected pediatric emergency patients, and typically children in these EDs are young with a median age 2 to 7 years in studies including children up to 13 or 18 years.^25,27^ Interestingly, several pediatric studies reported that boys are considerably overrepresented: in Pakistan 60% were boys,^22^ a study from Malaysia reported male to female ratio of 1.5:1,^24^ a Cambodian study reported 54% boys,^27^ and a large study from India reported a male to female ratio of 3:1.^23^ It is noteworthy that recent report^28^ from the COVID19 epidemic in China also indicates over representation of males among hospitalized cases that may indicate a sex-related susceptibility to serious infections, related to male sex. Few suggestions to explain this has been forwarded in the mentioned literature. Singhi et al^23^ suggested that a possible explanation could be increased vulnerability of boys to ill health. Another explanation may be that boys are more valued in these societies and, therefore, receive preferential attention from the family during illnesses, possibly particularly when resources are limited. In our study, 63% of the ED population were boys, while statistics from the Nepali population for 2017 shows that approximately 51% were boys in age groups 0 to 9 years and there were no meaningful sex-difference in age 10 to 19 years.^29^ It has been suggested that in Nepal, boys are more valued than girls and receive advantageous attention and are prioritized for health care during illness,^20,30,31^ but there are also possible roles of biological sex-differences in disease vulnerability. Interestingly, different studies have reported different sex-specific patterns for specific diseases. A study from Mauritania in children <5 years with diarrhea and respiratory diseases^32^ found higher risk for diarrhea-associated deaths among females while respiratory disease-associated deaths were more common in males. The patterns for injuries is more consistent: several studies have shown that the proportion of injured pediatric patients is higher in males than in females.^22,24,26,33^ It is likely that higher injury numbers in boys is at least partly due to physical activity outside the household. On the contrary, girls are typically kept in more safe environments and are involved in household chores from the young age. The assumption of gender inequality in our study may also be strengthened by the observation of more girls than boys in the PC categories unconsciousness and poisoning, possibly indicating that girls were taken to hospital when they were critical or life threateningly sick. This observation is novel and needs to be replicated in larger studies and similar settings. Also, our findings for distribution of mortality after ED discharge may indicate higher mortality in girls.

### Disposition and Pediatric ED

In our study, the proportion of emergency care seeking children that were hospitalized was 37% compared to 9%, 51%, and 25% in studies in similar age groups from Pakistan,^22^ Cambodia,^27^ and Malaysia^24^ respectively. In two other studies including younger pediatric populations from Pakistan^25^ and India^23^ the proportions of hospitalization were higher, (45%) and (42%) respectively. The high proportion of direct discharge home from ED in LMICs are in contrast with countries where the primary health care system screens the majority of patients before they eventually present to the hospital ED.^34^ Non-pediatric EDs in LMICs are typically crowded and receive patients for assessment with a wide spectrum of severity. Hence, pediatric competence and systems that can specifically evaluate and prioritize pediatric patients seem to be essential. The WHO conducted a study in 21 hospitals in low income settings to assess quality of care for seriously ill children,^35^ and found heterogeneity in pediatric critical care and competence in assessing pediatric illnesses and treatment. The main recommendations for improvements in LMIC pediatric emergency care include systematic triage, regular monitoring of patients, and strengthen pediatric emergency care competence.^4,35-37^ A review of emergency medicine in Nepal concludes that the country has critical shortage of emergency health service providers, and that the field of emergency medicine, and in particular pediatric emergency care, has largely been neglected in terms of health system development and specialist training.^10^

### Pediatric Triage in LMIC

Various triage systems have been developed to help emergency health care providers to make accurate priority decisions. The RETTS triage system was developed in Sweden and has been increasingly used in the Scandinavian countries.^17-19^

Pediatric emergency triage system was recently systematically assessed,^38^ and only one of the 18 studies of triage system reliability identified in the systematic review^38^ was from a LIC.^39^ Thus, the present report of triage priority categories in a pediatric population in a low income setting is unique. We also report associations between triage category and indicators of disease severity such as hospital admittance, ICU treatment and post-discharge mortality. We have identified no previous study that has assessed whether PPDM could be associated with severity at presentation. Further studies are needed to identify triage systems that can operate well in low-resource settings. It will be important to validate such systems.

### Pediatric Post-Discharge Mortality

PPDM in LIC was recently reviewed.^12^ In that systematic review of 24 individual studies, post-discharge mortality was often higher than in-hospital mortality. In our study, in-hospital mortality was less than 1%, and PPDM was two times higher. In previous studies, PPDM varied between 1% and 2% for anemia and malaria subpopulations and higher for those with malnutrition and pneumonia (3%-20%).^12^ Our study is based on an unselected emergency population, where more than half of the children were discharged without hospitalization and this can explain the low hospital mortality. It is important to note that the great majority of the studies included are from Africa, typically including only specific diagnostic groups.^12^ Only two of the studies,^40,41^ both from Africa, included all hospital admissions. Ours, however, assessed an emergency population where <40% were admitted. In those studies, infant age was associated with higher mortality than higher ages and this is in line with our findings. In-addition, PPDM was higher in females than males. Similar sex-specific analyses were only performed in few previous studies, and the patterns are not conclusive. Higher PPDM for girls (about two times than for boys) were found in a diarrheal study from Bangladesh,^42^ and for respiratory tract infections from Kenya.^43^ In our study, girls were under represented among the children seeking emergency care although the mortality was higher in girls. However, present findings related to triage categories do not support a hypothesis that girls were more severely sick at admittance to ED attendance (data not shown).

In our study, we found that post discharge mortality in children with infections was about 3-fold that of all other complaints combined. A recent study has reported that of the total sepsis deaths in children <5 years, 37.5% of deaths were due to respiratory infection and diarrheal disease.^8^ In other studies, specific diagnosis has been main target for the analyses.^12,41,44^ Thus, in those studies, typically malnutrition and respiratory tract infections had the highest PPDM risk. Importantly, inherent factors related to the health care in the hospital, such as overcrowding, experience with pediatric patients and factors related to costs of hospital stays may contribute to PPDM.

We explored the usefulness of triage codes in indicating risk of PPDM and found a very strong association, were PPDM in the red category was 16% and with a gradual reduction in risk by lower triage category. However, confidence intervals were wide indicating that the study has insufficient power to reject chance as an alternative explanation. We are not aware of other studies that have assessed triage and PPDM. In the reviewed post-discharge mortality studies, lower oxygen saturation on admission was associated with higher mortality risk after discharge^40,45^ but hypoxemia (<90%) was not associated with post-discharge mortality in malnourished patients.^46^

The present study assessed factors related to socioeconomic conditions and housing and we found that PPDM was higher for those that lived in rural compared to urban areas. We also found that living in a house with traditional stove was a strong indicator of higher mortality risk. Houses with traditional wooden stoves are typical for rural areas and low income and could also relate to low indoor temperatures and high smoke exposure, that in turn, could negatively influence specifically respiratory health. Our finding is supported by a meta-analysis of 24 studies by Dherani et al^47^ that concluded increased risk of pneumonia in young children by exposure to unprocessed solid fuel by a factor of 1.8.

## Conclusion

The study is observational, and the follow-up was not powered to reach firm conclusions on mortality. Nevertheless, our findings in this unselected pediatric emergency population highlighted that pediatric emergency patients typically present with infections and injuries, and that less girls than boys presented to ED. We found that high triage priority levels at ED presentation was strongly associated with need of advanced care and indicated higher mortality risk after discharge. These findings support that pediatric triage assessment systems can be implemented in a general ED in a low resource settings. The study indicates need for further follow-up studies on mortality in children who seek emergency care. We suggest that such interventions should emphasize effective treatment of infections and injuries, and identification of children at risk for complications after discharge.

Interventions to reduce emergency disease burden in children in low resource settings should emphasize prevention and effective treatment of infections and injuries.

## Supplemental Material

Supplementary_Material – Supplemental material for Pediatric Patients in a Local Nepali Emergency Department: Presenting Complaints, Triage and Post-Discharge Mortality Click here for additional data file. Supplemental material, Supplementary_Material for Pediatric Patients in a Local Nepali Emergency Department: Presenting Complaints, Triage and Post-Discharge Mortality by Samita Giri, Tine Halvas-Svendsen, Tormod Rogne, Sanu Krishna Shrestha, Henrik Døllner, Erik Solligård and Kari Risnes in Global Pediatric Health
