# Supplementary material for: Pediatric Patients in a Local Nepali Emergency Department: Presenting Complaints, Triage and Post-Discharge Mortality
Source: Glob Pediatr Health. 2020 Sep 18;7:2333794X20947926. doi: 10.1177/2333794X20947926 (PMC7502999; doi:10.1177/2333794X20947926)
Supplement: Supplementary_Material – Supplemental material for Pediatric Patients in a Local Nepali Emergency Department: Presenting Complaints, Triage and Post-Discharge Mortality [file Supplementary_Material.pdf]

Supplementary Material

1 Supplementary: List of variables at emergency department (ED)

1. Study Number
2. Hospital Number
3. Date of Admittance (DD/MM/YR)
4. Age (Years)
5. Sex (M/F)
6. Ethnicity
7. Time of Presentation
8. Temperature (Celsius)
9. Pulse (Beats per minute)
10. Respiration rate
11. Systolic Blood Pressure
12. Diastolic Blood Pressure
13. Oxygen Saturation
14. Glasgow Coma Scale (GCS)
15. Triage done (Yes/No)
16. Triage Code (Red/Orange/yellow/green)
17. Time of Triage
18. Time of Treatment
19. Presenting Complaint
20. Action in Emergency Room (Medication, Fluids, Oxygen, Antibiotics etc.)
21. Transfer to (General wards, ICU, OT)
22. ED disposition (Admitted, Discharged, LAMA/DOR, Referred)
23. Death (Yes/No)
24. Diagnosis at Discharge

2 Supplementary: Telephone interview guide at 90 days of ED disposition

1. Study Number
2. Hospital Number
3. Date of Interview
4. Visited Dhulikhel Hospital (DH) for same condition after the last visit (Yes/No)
5. Visited other health facility for same condition after the last visit to DH (Yes/No)
6. How long did you wait in ED before seeing a doctor (in minutes)
7. How do you like the services/patient management provided from ED (Very poor, Poor, OK/Fair, Good, very Good)
8. Health status now (Better, Unchanged, Worse, Died)
9. If died, Date of dead (DD,MM,YR)
10. Information given by (Patient, Relatives, Others)
11. a) In Labor case, if baby alive (Yes/No)
11. b) Number of baby delivered
12. Number of members in house living together
13. Main occupation
14. Education (Literate/Illiterate)
15. Completed education level
16. Smoking Tobacco (Yes/No)
17. If smoking tobacco, number per day
18. Fuel for Cooking (traditional cooking stove, improved cooking stove, gas stove, electricity)
19. Additional Information

3 Supplementary descriptions

3.1 Presenting Complaints Classification

The presenting complaints were recorded by an emergency nurse in the emergency form. The presenting complaint was recorded in free text format (for example; a lady with chest pain and fever). The complaints were retrospectively classified into seven main categories. The process of categorizations was performed in three phases using STATA software. In the first phase, in total 761"presenting complaint texts" (e.g. fever, chest pain, abdominal pain, limb fracture etc.) were generated and scored as 0 (no) or 1 (yes). In the second phase one or more "International Classification of Primary Care-2 (ICPC-2)" codes, were allocated to each patient based on the complaint categories first generated. In the third phase, one main presenting complaint was identified for each patient. Obvious related complaints were combined into one single group (e.g. fever and chills). The categories of seven main presenting complaints were; injuries, suspected infections, abdominal complaints, respiratory complaints, poisoning, unconsciousness and seizures, and other complaints. These categories were arranged in hierarchical order as shown in Figure 1A ("injuries" at first and "other complaints" at last; i.e. if a patient had injury irrespective to other complaints then he/she will be in injury category). Suspected infections complaint category included infections and also when presenting complaint was fever with no specification of organ system involved. The category "other complaints" included musculoskeletal, neurology, urinary, psychological and other complaints (general pain, eye/ear complaints etc.).

3.2 Follow-up interviews

Two trained research nurses conducted a structured telephone interview 90 days following the ED visit. The research nurse contacted all patients (n=2951) who provided phone numbers during the ED disposition. The research nurse contacted twice when patients were not reachable the first time. The telephone interview included information on 90-days mortality and exposure level to smoke.

4. Supplementary Tables

Table S1: Categories of presenting complaints and use of ICPC-2 codes

ICPC complaint Categories	Included ICPC-2 Codes
**Poisoning**	A86
**Injuries**	
Fall Injury	A80_b*
Transport Accident	A80_a*
Physical Assault	Z25
Bite	S12, S13
Burn	S14
Other Injury	A88, N80, D79, D80, R87, Y80, H76, H78, H79, F76, F79,
**Infections**	
Gastrointestinal Infection	D70
Urinary Infection	U71
Respiratory Infection	R74, R75, R76, R78, R81, R83, A70
Fever (only)	A02, A03
Other Infection	A78, A87, B70, N71, N73, S11
**Unconsciousness & Seizure**	A06, N07
**Cardiovascular (CVD)**	
CVD complaints	A11/K01, K04, K07
CVD disease	K71, K73, K86
**Respiratory complaints**	
Short Breath	R02
Asthma	R96
Other Respiratory	R03, R04, R05, R06, R07, R21, R24, R29, R99
**Gynaecology complaints**	
Pregnancy and Childbearing Complications	W03, W05, W29
Menstruation Complaints	X02, X06, X07
Female Genital Complaints	X01, X14, X21, X29, X77
**Abdomen and Digestive**	
Nausea, Vomiting & Diarrhoea	D09, D10, D11
Abdomen Pain	D01, D02, D06, D12, D25, D88
Other Digestive	D08, D13, D14, D15, D16, D17, D19, D20, D21, D24,
**Other Complaints**	
Urinary	U01, U02, U04, U05, U06, U07, U08, U95, U99
Neurology	N01, N05, N06, N08, N17, N19, N28, N29, N85, N88, N89
Musculoskeletal	L01, L02, L03, L05, L07, L09, L12, L14, L17, L18, L19,
Psychosocial Complaints	P04, P16, P19, P20, P29, P85, P86, P99
Other Complaints, NOS	A01, A04, A05, A08, A10, A16, A29, A92, A93, A94,

A80_b-Fall Injuries, A80_a-Road Transport Accidents, A80_c-Animal Injuries, A66-Referred to study hospital for intensive care unit.*New codes that does not comply with ICPC-2 codes.

Table S2. Characteristics of total patients (n=6317) and included patients (n=5740) in the analysis of Table1A

Characteristics	Total patients	Not Included	Included
**Total Patients, n (%)**	**6317**	**577**	**5740**
**Age, median (IQR)***	7 (2-12)	8 (4-12)	7 (2-12)
**Age (years), n (%)**			
<1	544 (9)	23 (4)	521 (9)
1-<5	1871 (30)	131 (23)	1740 (30)
5-16	3862 (61)	383 (66)	3479 (61)
Information NA	40 (1)	40 (7)	0
**Female, n (%)**	2337 (37)	227 (39)	2110 (37)
**Patient location, n(%)**			
Rural	3662 (58)	140 (24)	3522 (61)
Urban	1767 (28)	58 (10)	1709 (30)
Information NA	888 (14)	379 (66)	509 (9)
**Ethnicity, n (%)**			
Brahmin and Chhetri	2828 (45)	255 (44)	2573 (45)
Janajati	2797 (44)	251 (44)	2546 (44)
Dalit	548 (9)	41 (7)	507 (9)
Other	144 (2)	30 (5)	114 (2)
**Presenting Complaints, n(%)**			
Injuries	2284 (36)	7 (1)	2277 (40)
Suspected Infections	2256 (36)	19 (3)	2237 (39)
Abdominal complaints	453 (7)	2 (0.4)	451 (8)
Respiratory complaints	257 (4)	3 (1)	254 (4)
Poisoning	89 (1)	1 (0.2)	88 (2)
Unconsciousness and Seizures	86 (1)	4 (1)	82 (1)
Other complaints	375 (6)	24 (4)	351 (6)
Information NA	517 (8)	517 (90)	0

*Within available information. IQR=Interquartile range. NA=Information not available.

Table S3. Characteristics of total patients (n=5740) and included patients (n=4296) in the analysis of Table1B

Characteristics	Total patients	Not Included	Included
**Total Patients, n (%)**	**5740**	**1444**	**4296**
**Age, median (IQR)**	7 (2-12)	6 (2-12)	7 (2-12)
**Age (years), n (%)**			
<1	521 (9)	155 (11)	366 (9)
1-<5	1740 (30)	465 (32)	1275 (30)
5-16	3479 (61)	824 (57)	2655 (62)
**Female, n (%)**	2110 (37)	550 (38)	1560 (36)
**Patient location, n(%)**			
Rural	3522 (61)	845 (59)	2677 (62)
Urban	1709 (30)	389 (27)	1320 (31)
Information NA	509 (9)	210 (15)	299 (7)
**Ethnicity, n (%)**			
Brahmin and Chhetri	2573 (45)	643 (45)	1930 (45)
Janajati	2546 (44)	659 (46)	1887 (44)
Dalit	507 (9)	114 (8)	393 (9)
Other	114 (2)	28 (2)	86 (2)
**Presenting Complaints, n(%)**			
Injuries	2277 (40)	537 (37)	1740 (41)
Suspected Infections	2237 (39)	578 (40)	1659 (39)
Abdominal complaints	451 (8)	108 (7)	343 (8)
Respiratory complaints	254 (4)	67 (5)	187 (4)
Poisoning	88 (2)	28 (2)	60 (1)
Unconsciousness and Seizures	82 (1)	22 (2)	60 (1)
Other complaints	351 (6)	104 (7)	247 (6)

IQR=Interquartile range. NA=Information not available.

Table S4. Characteristics of total patients (n=5740) and interviewed patients (n=961) included in the analysis of Table2

Characteristics	Total patients	Not Included	Included
**Total Patients, n (%)**	**5740**	**4779**	**961**
**Age, median (IQR)**	7 (2-12)	7 (2-12)	7 (2-12)
**Age (years), n (%)**			
<1	521 (9)	425 (9)	96 (10)
1-<5	1740 (30)	1465 (31)	275 (29)
5-16	3479 (61)	2889 (60)	590 (61)
**Female, n (%)**	2110 (37)	1770 (37)	340 (35)
**Patient location, n(%)**			
Rural	3522 (61)	2874 (60)	648 (67)
Urban	1709 (30)	1396 (29)	313 (33)
Information NA	509 (9)	509 (11)	0
**Ethnicity, n (%)**			
Brahmin and Chhetri	2573 (45)	2128 (45)	445 (46)
Janajati	2546 (44)	2130 (45)	416 (43)
Dalit	507 (9)	413 (9)	94 (10)
Other	114 (2)	108 (2)	6 (1)
**Presenting Complaints, n(%)**			
Injuries	2277 (40)	1911 (40)	366 (38)
Suspected Infections	2237 (39)	1857 (39)	380 (40)
Abdominal complaints	451 (8)	361 (8)	90 (9)
Respiratory complaints	254 (4)	198 (4)	56 (6)
Poisoning	88 (2)	75 (2)	13 (1)
Unconsciousness and Seizures	82 (1)	73 (2)	9 (1)
Other complaints	351 (6)	304 (6)	47 (5)

IQR=Interquartile range. NA=Information not available.

Table S5. Characteristics of total patients (n=5740) and triaged patients (n=1248) included in the analysis of Table 3A

Characteristics	Total patients	Not Included	Included
**Total Patients, n (%)**	**5740**	**4492**	**1248**
**Age, median (IQR)**	7 (2-12)	7 (2-12)	7 (2-13)
**Age (years), n (%)**			
<1	521 (9)	411 (9)	110 (9)
1-<5	1740 (30)	1379 (31)	361 (29)
5-16	3479 (61)	2702 (60)	777 (62)
**Female, n (%)**	2110 (37)	1644 (37)	466 (37)
**Patient location, n(%)**			
Rural	3522 (61)	2702 (60)	820 (66)
Urban	1709 (30)	1323 (29)	386 (31)
Information NA	509 (9)	467 (10)	42 (3)
**Ethnicity, n (%)**			
Brahmin and Chhetri	2573 (45)	2033 (45)	540 (43)
Janajati	2546 (44)	1967 (44)	579 (46)
Dalit	507 (9)	396 (9)	111 (9)
Other	114 (2)	96 (2)	18 (1)
**Presenting Complaints, n(%)**			
Injuries	2277 (40)	1771 (39)	506 (41)
Suspected Infections	2237 (39)	1763 (39)	474 (38)
Abdominal complaints	451 (8)	348 (8)	103 (8)
Respiratory complaints	254 (4)	195 (4)	59 (5)
Poisoning	88 (2)	68 (2)	20 (2)
Unconsciousness and Seizures	82 (1)	62 (1)	20 (2)
Other complaints	351 (6)	285 (6)	66 (5)

IQR=Interquartile range. NA=Information not available.

Table S6. Characteristics of total triaged patients (n=1462) and those included interviews (n=497) that are included in the analysis of Table 4

Characteristics	Total patients	Not Included	Included
**Total Patients, n (%)**	**1462**	**965**	**497**
**Age, median (IQR)**	7 (2-13)	7 (2-13)	8 (2-12)
**Age (years), n (%)**			
<1	135 (9)	87 (9)	48 (10)
1-<5	420 (29)	294 (30)	126 (25)
5-16	907 (62)	584 (61)	323 (65)
**Female, n (%)**	550 (38)	366 (38)	184 (37)
**Patient location, n(%)**			
Rural	972 (66)	636 (66)	336 (68)
Urban	442 (30)	281 (29)	161 (32)
Information NA	48 (3)	48 (5)	0
**Ethnicity, n (%)**			
Brahmin and Chhetri	630 (43)	397 (41)	233 (47)
Janajati	676 (46)	459 (48)	217 (44)
Dalit	135 (9)	89 (9)	46 (9)
Other	21 (1)	20 (2)	1 (0.2)
**Presenting Complaints, n(%)**			
Injuries	575 (39)	395 (41)	180 (36)
Suspected Infections	571 (39)	366 (38)	205 (41)
Abdominal complaints	120 (8)	72 (7)	48 (10)
Respiratory complaints	66 (5)	41 (4)	25 (5)
Poisoning	24 (2)	17 (2)	7 (1)
Unconsciousness and Seizures	23 (2)	18 (2)	5 (1)
Other complaints	83 (6)	56 (6)	27 (5)

IQR=Interquartile range. NA=Information not available.

Table S7. Characteristics of patients before earthquakes and after earthquakes

Characteristics	Total	Before EQ	After EQ
**Total Patients, n (%)**	**5740**	**2239 (39)**	**2501 (61)**
**Age, median (IQR)**	7 (2-12)	7 (3-12)	6 (2-12)
**Age (years), n (%)**			
<1	521 (9)	115 (5)	406 (12)
1-<5	1740 (30)	685 (31)	1055 (30)
5-16	3479 (61)	1439 (64)	2040 (58)
**Female, n (%)**	2110 (37)	814 (36)	1296 (37)
**Patient location, n(%)**			
Rural	3522 (61)	1298 (58)	2224 (64)
Urban	1709 (30)	611 (27)	1098 (31)
Information NA	509 (9)	330 (15)	179 (5)
**Ethnicity, n (%)**			
Brahmin and Chhetri	2573 (45)	1082 (48)	1491 (43)
Janajati	2546 (44)	942 (42)	1604 (46)
Dalit	507 (9)	161 (7)	346 (10)
Other	114 (2)	54 (2)	60 (2)
**Presenting Complaints, n(%)**			
Injuries	2277 (40)	917 (41)	1360 (39)
Suspected Infections	2237 (39)	877 (39)	1360 (39)
Abdominal complaints	451 (8)	158 (7)	293 (8)
Respiratory complaints	254 (4)	97 (4)	157 (4)
Poisoning	88 (2)	31 (2)	57 (2)
Unconsciousness and Seizures	82 (1)	30 (1)	52 (2)
Other complaints	351 (6)	129 (6)	222 (6)

EQ=Eathquake period. IQR=Interquartile range. NA=Information not available.
